# Complete Genomic Analysis of *Enterococcus faecium* Heat-Resistant Strain Developed by Two-Step Adaptation Laboratory Evolution Method

**DOI:** 10.3389/fbioe.2020.00828

**Published:** 2020-07-23

**Authors:** Bonggyu Min, DongAhn Yoo, Youngho Lee, Minseok Seo, Heebal Kim

**Affiliations:** ^1^Department of Agricultural Biotechnology, and Research Institute of Agriculture and Life Sciences, Seoul National University, Seoul, South Korea; ^2^Interdisciplinary Program in Bioinformatics, Seoul National University, Seoul, South Korea; ^3^Department of Computer Convergence Software, Korea University, Sejong, South Korea; ^4^C&K Genomics Inc., Seoul, South Korea

**Keywords:** *Enterococcus faecium*, adaptive laboratory evolution, heat resistance, thermal critical point, genomic analysis, fatty acid composition, probiotics animal feed

## Abstract

Stress resistance is an important trait expected of lactic acid bacteria used in food manufacturing. Among the various sources of stress, high temperature is a key factor that interrupts bacterial growth. In this regards, constant efforts are made for the development of heat-resistant strains, but few studies were done accompanying genomic analysis to identify the causal factors of the resistance mechanisms. Furthermore, it is also thought that tolerance to multiple stresses are equally important. Herein, we isolated one *Enterococcus faecium* strain named BIOPOP-3 and completed a full-length genome sequence. Using this strain, a two-step adaptive laboratory evolution (ALE) method was applied to obtain a heat-resistant strain, BIOPOP-3 ALE. After sequencing the whole genome, we compared the two full-length sequences and identified one non-synonymous variant and four indel variants that could potentially confer heat resistance, which were technically validated by resequencing. We experimentally verified that the evolved strain was significantly enhanced in not only heat resistance but also acid and bile resistance. We demonstrated that the developed heat-resistant strain can be applied in animal feed manufacturing processes. The multi-stress-resistant BIOPOP-3 ALE strain developed in this study and the two-step ALE method are expected to be widely applied in industrial and academic fields. In addition, we expect that the identified variants which occurred specifically in heat-resistant strain will enhance molecular biological understanding and be broadly applied to the biological engineering field.

## Introduction

*Enterococci* are the symbiotic lactic acid bacteria (LAB) in gastrointestinal tract of human and other animals (Murray, 1990). This genus has been actively investigated for its potential as probiotic bacteria. *Enterococci* as probiotics are known to prevent or treat diarrhea in livestock and pets (Franz et al., 2011). *Enterococcus faecium* (*E. faecium*) SF68 has been successfully applied in the treatment of diarrhea to help reduce symptoms and normalize bowel movements in children and adults (Buydens and Debeuckelaere, 1996). In the past few years, numerous benefits of probiotics have been discovered, garnering increased commercial interest of probiotic strains in various products (Gueimonde and Sánchez, 2012). However, most probiotics in use are sensitive to diverse environmental stresses. The stability of the probiotics can be affected by different stresses coming from manufacturing and storing of the product, such as temperature, pH, and etc. (Gueimonde et al., 2012). *Enterococci* are generally known to exhibit high viability under various types of stress such as high salinity and low pH, and hence they are considered suitable in various industries. Based on these advantages, *Enterococci* are widely applied for fermentation of foods (Ogier and Serror, 2008; Martín-Platero et al., 2009; Franz et al., 2011). *E. faecium* is frequently found in raw milk and dairy products and plays an important role in cheese-making and other food fermentation processes (Yerlikaya and Akbulut, 2020). Several *E. faecium* strains such as HY07 and T110 are known to provide health benefits to the host and are therefore used as commercial probiotics (Natarajan and Parani, 2015; Duan et al., 2019). However, some *Enterococcus* strains have putative virulence factors such as gelatinase, cytolysin, and endocarditis antigens (Vankerckhoven et al., 2004; Martín-Platero et al., 2009). Also, vancomycin-resistant *enterococci* (VRE) are characterized by their multidrug-resistance to commonly used antibiotics (Ahmed and Baptiste, 2018). Therefore, it is very important to ensure that *enterococci* have no such risks before one could use them as a probiotics.

During manufacturing, bacteria are often subjected to severe environmental stress including high temperature (Zhou et al., 2019). Manufacturing processes such as pasteurization often require high temperatures (>60°C), which can be lethal to bacteria (Torres-Maravilla et al., 2016). Thus, development of heat-tolerant strains is a major issue in bioprocesses. Adaptive Laboratory Evolution (ALE) could provide an efficient solution for this case. ALE is a method that relies on the basic mechanisms of molecular evolution that accumulate in microbial populations (Dragosits and Mattanovich, 2013). During ALE experiment, microorganisms are exposed to a stressful condition for a long time, allowing us to select for the population with the improved phenotypes (Du et al., 2020). ALE is being successfully utilized for a variety of stress such as acid (Fletcher et al., 2017), osmotic pressure (Dhar et al., 2011), and temperature (Chen et al., 2015; Deatherage et al., 2017). In addition, other studies improved on antibiotic resistance of *Lactobacillus plantarum* through ALE (Dong et al., 2019; Feng et al., 2019). These conventional methods for improving the stress resistance involve repeated culturing of microbial population over a long period of time under a particular stress condition (Rudolph et al., 2010; Oide et al., 2015; Wang et al., 2017; Kwon et al., 2018).

Adaptive laboratory evolution has been applied to isolate heat-resistant mutants of well-characterized microorganisms such as *Escherichia coli* and *Saccharomyces cerevisiae* allowing the resulting population to overcome their growth temperature limits of over 40°C (Rudolph et al., 2010; Caspeta et al., 2014; Sandberg et al., 2014). However, there are few cases in which ALE for improving heat resistance was applied to LAB such as *E. faecium*. In this study, we applied ALE to develop a robust heat-resistant *E. faecium* that can survive under high temperature. First, we isolated BIOPOP-3 wild-type (WT) from fermented dairy product, generated whole genome data and completed genomic analysis to identify its genomic features. We investigated the thermal threshold of BIOPOP-3 strain and conducted a modified ALE method to improve the stress resistance of *E. faecium* BIOPOP-3. Additional experiments were also performed to investigate whether the resulting strain (BIOPOP-3 ALE) is resistant to other stresses. Finally, a genomic analysis was performed to find the molecular biomarker that confers heat resistance by comparing the genome sequence of BIOPOP-3 ALE strain with BIOPOP-3 WT.

## Materials and Methods

### Bacterial Culture and Strain Identification

The probiotic strain BIOPOP-3 was isolated from a fermented dairy product in South Korea. They were cultured anaerobically at 37°C in sterile deMan Rogosa Sharpe (MRS, Difco, Becton Dickinson Co., Sparks, MD, United States) and incubated at 37°C for 24 h. The bacterial cells were stored at −80°C as stock solution in 40% glycerol. The 16S rRNA gene was sequenced by Macrogen Inc. (Seoul, South Korea). The genomic DNA was extracted according to the instruction provided by the manufacturer of DNA extraction kit (QIAGEN, United States) (Guo et al., 2016) and the 16S rRNA gene was amplified using the universal bacterial primer sets: 27F 5’ (AGA GTT TGA TCM TGG CTC AG) 3’ and 1492R 5’ (TAC GGY TAC CTT GTT ACG ACT T) 3’ (Devereux and Willis, 2011). The NCBI Basic Local Alignment Search Tool (BLAST) was used to assign taxonomy (Altschul et al., 1990). The phylogenetic tree was built using the MEGA-X software (Kumar et al., 2018).

### Whole Genome Sequencing of BIOPOP-3 Strains

The genomic DNA was extracted and purified using an UltraClean Microbial DNA Isolation Kit (MoBio, Carlsbad, CA, United States) according to the manufacturer’s protocols. NanoDrop spectrophotometer (Thermo Scientific, Wilmington, DE, United States) was used to measure the concentration and purity of extracted genomic DNA. DNA library construction was performed using the Pacific Biosciences (PacBio) SMRT platform based on the standard protocol. Raw sequence data from the PacBio Sequel II system were filtered and assembled using the PacBio SMRTLink v7.0, *de novo* Assembly (HGAP 4) (Biosciences, 2019). In the assembly process, genome size parameter was set to 3Mbp and other parameters were set to default. Assembled contigs were filtered for the length of < 20,000 bp and the mean depth coverage of 1,330× was observed. An iterative polishing process was conducted for the pre-assembled genome sequences, until no genomic variants were identified. To identify genetic changes in the evolved mutant, whole genome sequence of the BIOPOP-3 ALE was compared with that of BIOPOP-3 WT.

Also, to technically verify the variants, resequencing of BIOPOP-3 ALE sequence data was performed using BIOPOP-3 WT as the reference genome via SMRTlink v.7.0 resequencing service.

### Annotation of Genomic Features

The genome sequence was annotated with Rapid Annotation using Subsystem Technology (RAST) (Aziz et al., 2008). tRNA and rRNA genes from the genomes were predicted using tRNA_scan-SE (Lowe and Eddy, 1996) and RNAmmer (Lagesen et al., 2007), respectively. PlasmidFinder was used to search for plasmids within the genome (Carattoli et al., 2014). Prophage sequences were predicted and annotated using PHASTER (Arndt et al., 2016) and bacterial insertion elements were identified by ISfinder (Siguier, 2006). The clustered regularly interspaced short palindromic repeats (CRISPR) regions were predicted using CRISPRCasFinder (Couvin et al., 2018). Genomic islands (GIs) were predicted using IslandViewer4 (Bertelli et al., 2017). Bacteriocin and secondary metabolite biosynthetic clusters were identified using BAGEL4 (Van Heel et al., 2018) and antiSMASH version 5.1.1 (Blin et al., 2019), respectively. VirulenceFinder 2.0 was used to determine the presence of virulence factors in the genome (Joensen et al., 2014) and antibiotic resistance genes were predicted using the comprehensive antibiotic resistance database (CARD) (McArthur et al., 2013). The map of the genome was generated using the CGview server (Grant and Stothard, 2008).

### Comparative Genomic Analysis

A total of 111 complete genome sequences of *E. faecium* strains were downloaded from the NCBI database^1^. Calculation of BLASTN-based average nucleotide identity (ANI) was carried out using pyani (Pritchard et al., 2016). An approximate maximum-likelihood phylogenetic tree was constructed and visualized using MEGA-X software (Kumar et al., 2018). Functional annotation was performed by Cluster of Orthologous Group (COG) database (Tatusov, 2000). The pan/core genome analysis was performed using Roary (Page et al., 2015) with the cut-off of 90% BLASTp percentage identity. ORF content of each chromosome was classified into functional gene clusters using the gene family method.

### Procedure of Two-Step Adaptive Laboratory Evolution

The evolution was initiated with one *E. faecium* BIOPOP-3 WT strain. Before the experiment, a stock of WT strain was thawed at room temperature and streaked on MRS agar plate. This plate was incubated at 37°C for 48 h. One single colony was transferred to 10 ml of MRS medium and incubated at 37°C for 24 h. As a first step of ALE, 10 μl of sample was transferred to 1.5 ml micro tube with 990 μl MRS broth pre-heated at a test temperature and heat-treated in a dry bath for 1 min. Samples were cooled down for 5 min at room temperature, and incubated at 37°C for 24 h. This procedure was repeated for two more days. Heat treatment was performed starting from 60°C and the temperature was gradually increased by 3°C until the point where surviving strain was not detected. After confirming that the strain could not survive under the final treated temperature, the surviving strain before the final treated temperature was screened and was designated as heat-adapted strain. Next, to overcome thermal threshold of heat-adapted strain, we implemented the second step of ALE experiment. 10 μl culture of heat-adapted strain was transferred into a new 1.5 ml micro tube with 990 μl MRS broth pre-heated at 75°C. Using a dry bath, heat treatment was performed at 75°C for 1 min and incubated at 37°C for 24 h. This process was repeated for 25 days. During the above procedure, the survival ratio was recorded every 2 days. Samples of all steps were stored at -80°C in 40% glycerol as stocks. The evolved mutant of *E. faecium* was designated as BIOPOP-3 ALE.

### Evaluation of Heat Resistance Enhancement

Each stock of WT, heat-adapted, and ALE strains was thawed at room temperature and streaked on a plate. They were incubated at 37°C for 48 h and then each colony was isolated in 10 ml of sterile MRS and incubated at 37°C for 24 h. Heat treatment was performed in the dry bath starting from the initial temperature of 60°C to 87°C with the temperature increased by 3°C. At each temperature, 10 μl of bacterial cells were added into 990 μl of MRS broth and heated for 1 min. They were serially diluted with 0.85% saline and were spread on MRS agar plates followed by incubation of 48 h at 37°C. In addition, the 100 μl of the culture was added into 10 ml of pre-heated MRS broth and heated in a water bath at 60°C for 60 min (Shin et al., 2018). The survival ratio was recorded every 10 min. After the heat treatment, the broth was cooled down for about 5 min at room temperature. The broth containing the bacterial colony was diluted and spread on MRS agar plates followed by incubation of 48 h at 37°C. The *D*-value (decimal reduction time) was calculated following the equation:

t=D×(logN-0logN)f

where t: time (min), D: *D*-value at heat conditions, N_0_: initial concentration of microorganisms, Nf: final concentration of microorganisms (Mazzola et al., 2003). *D*-values for three different cultures were calculated as the negative inverses of the regression line slopes obtained by plotting the log number of survivors against time (Álvarez-Ordóñez et al., 2009). All experiments were conducted in triplicate. The survival ratio of each strain was determined by counting the number of colonies on the plates and dividing the colony forming units (CFUs) of heat treated culture by the CFUs of non-heat treatment (control) culture (Shibai et al., 2017). Two groups *t*-test was performed between ALE and others at critical point 75°C.

### Cross Protection Against Acid and Bile Stress

The cells were pre-cultured at 37°C for 24 h. After that, they were harvested by centrifugation (4,000 rpm, 10 min, 4°C) and washed twice with phosphate-buffered saline (PBS) adjusted to pH 7.0. To measure the resistance toward low pH, cell pellets were resuspended in PBS where the pH was adjusted to 2.0 and 3.0. Cell suspension was incubated for 3 h at 37°C. To evaluate their viability, they were diluted with 0.85% saline and were spread on MRS agar plates followed by incubation of 48 h at 37°C. Bile salt resistance of each strain was also examined. After harvested by centrifugation, cell pellets were washed twice and resuspended in PBS adjusted to concentrations of 0.5 and 1% with bile salts (cholic acid sodium salt 50% and deoxycholic acid sodium salt 50%, Sigma Aldrich, 48305). Cell suspension was incubated for 3 h at 37°C, diluted and spread on MRS agar plates for evaluation of viability before another incubation at 37°C for 48 h. All experiments were conducted in triplicate. The survival ratio was determined by counting the number of colonies on the plates and dividing the CFUs of the treated cultures by the CFUs of non-treated (control) cultures (Shibai et al., 2017). Hypothesis test was conducted based on the one-way ANOVA model.

### Membrane Fatty Acid Composition Analysis

The composition of cell membrane fatty acid was quantified using gas chromatography analysis outlined by Garces and Mancha (GarcÉs and Mancha, 1993). BIOPOP-3 WT, heat-adapted, and ALE strains were cultured in MRS broth and incubated at 37°C for 24 h. Cells were then harvested by centrifugation and washed twice with distilled water. Pellets were transferred to tubes with Teflon-lined caps and pentadecenoic acid (C15:0) was used as an internal standard. For lipid extraction, tubes were placed in a water bath at 80°C for 2 h. They were then cooled down at room temperature. After shaking and precipitating the sample, the contents were separated into two layers. The upper layer containing Fatty Acid Methyl Esters was extracted and analyzed using Agilent 7890A gas chromatography (Agilent, United States) equipped with a flame ionization detector and a DB-23 column (60 mm × 0.25 mm × 0.25 μm) (Agilent Technologies, Inc., Wilmington, DE, United States). The results were shown as relative percentages of each fatty acid and the ratios of saturated fatty acids and unsaturated fatty acids were calculated (Shin et al., 2018).

### Evaluation of Heat and Storage Stabilities of BIOPOP-3 Mixed With Animal Feed

The stock of ALE strain was thawed at room temperature and streaked on agar plate. After incubation of the plate at 37°C for 48 h, the isolated single colony of the plate was transferred into test tubes with 10 ml of MRS and incubated at 37°C for 24 h. The animal feed was mixed with broth type strain adjusted to contain only 1% of the total volume, and a bit of calcium carbonate (CaCO_3_). The wet and dry conditions were used during the heat treatment. Heating temperature were 65, 80, and 85°C and the duration of the heating were 1, 3, and 5 min at each temperature. The BIOPOP-3 ALE strain was transformed into powder using a freeze-dryer. The media contained 6% (w/v) skim milk as cryoprotectant. The mixture was frozen in a dryer at -40°C for the primary drying followed by the secondary drying in a stepwise decrease of temperature down to −85°C for a total of 24 h (Shin et al., 2018). The animal feed was mixed with the freeze-dried powder adjusted to contain 1% of the total volume, and calcium carbonate (CaCO_3_). After that, heat treatment was performed under the wet condition in the same procedure described above. To assess long-term storage stability, the animal feed was mixed with the lyophilized ALE strain adjusted to contain 1% of the total volume and stored at room temperature for 6 months. Survival ratio was recorded once a month. For evaluation of viability, the bacteria were diluted and spread on MRS agar plates. Under anaerobic condition, they were incubated at 37°C for 48 h. All experiments were conducted over three times. The survival ratio was calculated by counting the number of colonies on the plates and dividing the CFUs of the treated culture by the CFUs of non-treated (control) culture (Shibai et al., 2017).

### Accession Number

The complete sequences of *E. faecium* BIOPOP-3 WT and ALE are available in the NCBI database with accession numbers, PRJNA615057.

## Results

### Genomic Features of Isolated Wild-Type BIOPOP-3 Strain

**FIGURE 1:**
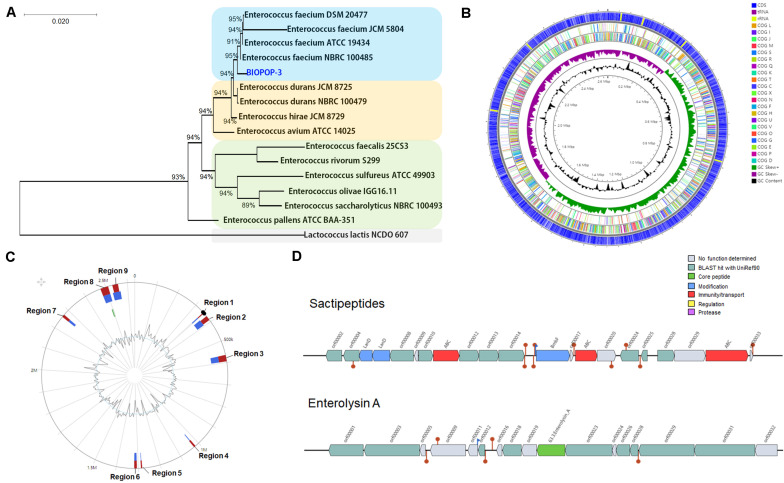
Genomic features of BIOPOP-3 strain. **(A)** Neighbor-joining tree constructed based on the 16S rRNA sequences. **(B)** Genomic structure of BIOPIP-3 strain. From outside to inner of the circle: CDS, the distribution of COG functional category, GC skewness, and percentage of GC contents. **(C)** Predicted nine genomic islands. **(D)** Predicted bacteriocin-related genes.

We isolated a BIOPOP-3 WT strain. The phylogenetic tree based on the 16S rRNA showed that BIOPOP-3 is close to *E. faecium* (Figure 1A) and the WT of BIOPOP-3 showed identity score of 99.53% with *E. faecium* HB-1 strain. The genome of BIOPOP-3 consisted of a complete circular genome sequence of 2,633,054 bp (38.4% GC contents) and one plasmid of 141,022 bp (35.8% GC contents). We annotated 2,670 predicted coding sequences (CDSs), 684 uncharacterized sequences, 69 tRNAs, and 18 rRNA for the circular chromosome (Figure 1B) and 178 predicted CDSs and 81 uncharacterized sequences for the plasmid. Two complete and three incomplete prophage regions were found in the chromosome of BIOPOP-3 (Supplementary Table 1). In complete phage regions, 40 and 24 phage-related genes were found, which encode for lysin, tail, capsid, head, portal, terminase, and integrase. From the incomplete phage regions, we identified the integrase and lysin encoding genes as well as five putative transposase genes. The BIOPOP-3 strain was compared to the closest relative *E. faecium* BM4105-RF to check if the phage regions are overlapping with any GI regions. Among the nine putative GI regions identified (Figure 1C), four regions overlapped with the prophage regions. In addition, various prophage-related genes were found in other regions. These genes were predicted as hypothetical genes or proteins-coding genes originated from bacteriophages. As CRISPR could play an important role in the interaction of bacteria and mobile genetic elements (Westra et al., 2012), we additionally identified CRISPR elements from the BIOIPOP-3 WT genome (Supplementary Table 2).

We identified a bacteriocin gene that is one of the important bacterial features. A genomic cluster encoding for sactipeptides was located at 1,007,495-1,027,495bp of the BIOPOP-3 chromosome (Figure 1D). Moreover, we found enterolysin A, a class III bacteriocin located at 68,856–89,399 bp of plasmid. We also identified six secondary metabolite-related gene clusters including Type III polyketide synthase (TIIIPKS) located at 1,975,268–2,016,422 bp of the chromosome (Supplementary Table 3). Finally, two genes functionally related to antibiotic resistance were identified (Supplementary Table 4). No virulence factor was detected in the genome of BIOPOP-3 strain.

### Comparative Genomic Analysis With Other *E. faecium* Strain

**FIGURE 2:**
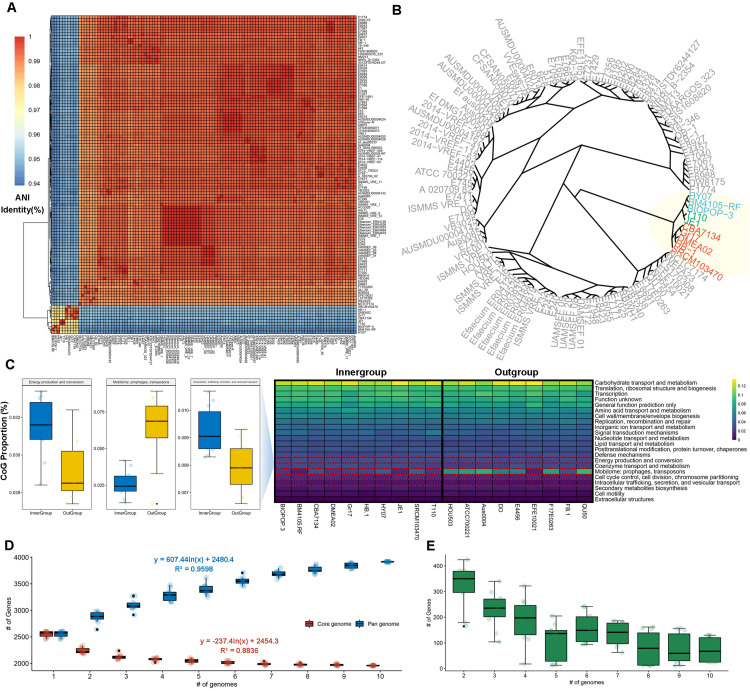
Comparative genomics with 112 *E. faecium* strains. **(A)** Hierarchical clustering based on ANI values of 112 *E. faecium* strains. **(B)** BIOPOP-3 WT strain was clustered with nine other *E. faecium* strains. **(C)** The three COG functional terms significantly differed between the inner-group containing BIOPOP-3 and the rest of *E. faecium* strains. **(D)** Pan and core genome estimates of 10 *E. faecium* strains in the inner-group containing BIOPOP-3. **(E)** The number of newly identified genes observed when new genomes are added sequentially.

We collected 111 available complete genome sequences of the *E. faecium* from the NCBI database to investigate its relationship with BIOPOP-3 WT strain. As a result of estimating the relationship between 112 *E. faecium* strains, we found that these strains are largely divided into two groups (Figure 2A). In case of BIOPOP-3 WT strain, it was found to cluster with nine other *E. faecium* strains, of which the most similar was the BM4105-RF (99.0%) and HY07 (98.68%) strains (Figure 2B). Suspecting there could be independent functional differences between the two clusters, we first examined 10 strains close to BIOPOP-3 (Table 1). We additionally investigated the difference in the COG functional category from the remaining *E. faecium* strains and found three significant functional terms at 5% significance level with multiple testing adjustment: category C (Energy production and conversion; P_adj_: 0.035), category X (Mobilome: prophages, transposons; P_adj_: 0.027), and category U (Intracellular trafficking, secretion, and vesicular transport; P_adj_: 0.035) (Figure 2C and Supplementary Table 5). We also examined exchange of genetic materials among BIOPOP-3 and other microbial populations through pan and core genome analysis with 9 strains similar to BIOPOP-3. As a result, the number of core genes was found to be about half of the pan gene (Figure 2D). Moreover, the number of newly added genes were counted to investigate whether the *E. faecium* pan-genome is open (Figure 2E). Based on the number of genes observed in 10 *E. faecium* genomes, we found that an *E. faecium* pan-genome is expected to contain 72 new genes on average, suggesting that 10 *E. faecium* strains, including BIOPOP-3 WT have the structure of an open pan-genome.

**TABLE 1:** Features of *E. faecium* strains close to BIOPOP-3.

**Strain**	**Chr. Length (Mbp)**	**CDS**	**tRNA**	**rRNA**	**Hypothetical protein (%)**	**Assigned function (%)**	**Prophage (Complete region)**	**Prophage (Incomplete region)**	**CRISPR**	**GC (%)**	**Plasmid (Size Kbp)**
BIOPOP-3	2.63	2670	69	18	25.6	74.4	2	3	1	38.4	1(141)
BM4105-RF	2.68	2640	68	18	25.2	74.8	1	0	3	38.3	-
CBA7134	2.63	2648	69	18	23.9	76.1	2	2	2	38.5	2(190, 20)
DMEA02	2.57	2556	68	18	23.2	76.8	0	3	2	38.5	1(170)
Gr17	2.59	3149	67	18	22	78	2	2	1	38.5	1(50)
HB-1	2.62	2648	70	18	23.2	76.8	3	1	1	38.5	1(150)
HY07	2.59	2563	67	18	23.6	76.4	0	2	3	38.3	2(20, 260)
JE1	2.72	2689	68	18	24.3	75.7	2	0	2	38.5	1(80)
SRCM103470	2.7	2746	70	18	25.3	74.7	3	5	3	38.3	2(240, 4)
T110	2.69	2666	65	18	24.2	75.8	2	1	2	38.5	1(40)

### Improvement of Heat Resistance for BIOPOP-3 WT Strain

**FIGURE 3:**
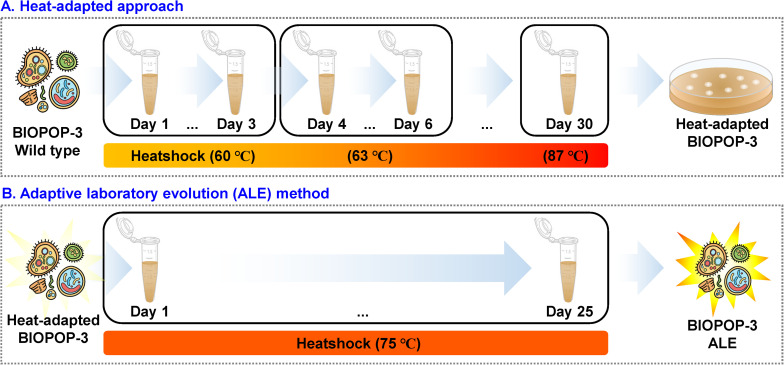
Procedure for adaptive laboratory evolution experiment. **(A)** The procedure of heat-adapted experiments. **(B)** The temperature was constant at 75°C. The duration of heat shock was 1 min and after heat shock, cells were cooled down at room temperature followed by incubation of 24 h at 37°C.

**FIGURE 4:**
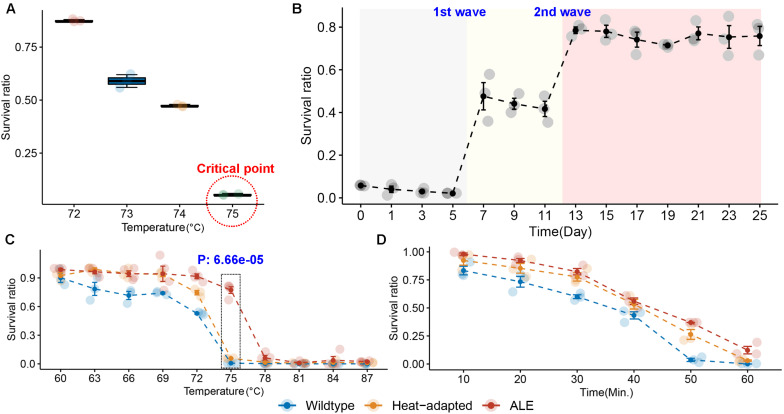
Results of experiments to overcome the heat resistance limits of BIOPOP-3 strain. **(A)** Identification of heat resistance limit of heat-adapted BIOPOP-3 strain. **(B)** Change in survival rate of BIOPOP-3 in an ALE experiment for 25 days. **(C)** Comparison of survival rates of BIOPOP-3 WT, heat-adapted, and ALE strains at different temperature conditions. The heat exposure was fixed at 1 min. *P*-value from two groups *t*-test between ALE and others at 75°C. **(D)** Comparison of survival rates of three strains at different heat exposure time. The temperature was fixed at 60°C.

We performed a two-step experiment to improve the heat resistance of the BIOPOP-3 WT strain isolated from fermented dairy product, resulting in BIOPOP-3 heat-adapted and BIOPOP-3 ALE (Figure 3). After the heat adaptation procedure, a search for the critical point for temperature revealed that the survival rate of BIOPOP-3 heat-adapted strain rapidly decreased at 75°C (Figure 4A). During the ALE procedure of 25 days, two major changes in the survival rate were observed on day 7 and day 13 (Figure 4B). Survival rate remained constant above 70% after day 13. The average survival rate observed on the last day was 75.85% which is significantly higher than the control strain by approximately 70%. Apart from that, improved heat resistance of BIOPOP-3 ALE was observed at all temperatures compared to WT and heat-adapted strains (Figure 4C) with the most significant difference being observed at 75°C (P: 6.66e-05). Even in experiments where the temperature condition was fixed at 60°C and the exposure time was a variable factor, BIOPOP-3 ALE outperformed in terms of survival rate compared to other strains (Figure 4D). We additionally calculated their heat resistance by *D*-value (decimal reduction time) which is exposure time required to cause one log_10_ or 90% reduction of the initial population under specified temperature (Mazzola et al., 2003). As a result, the WT strain showed the *D*-value of 5.7 while that of the ALE strain was improved to 6.6.

### Improved Resistance of BIOPOP-3 ALE Strain Toward Various Stresses

**FIGURE 5:**
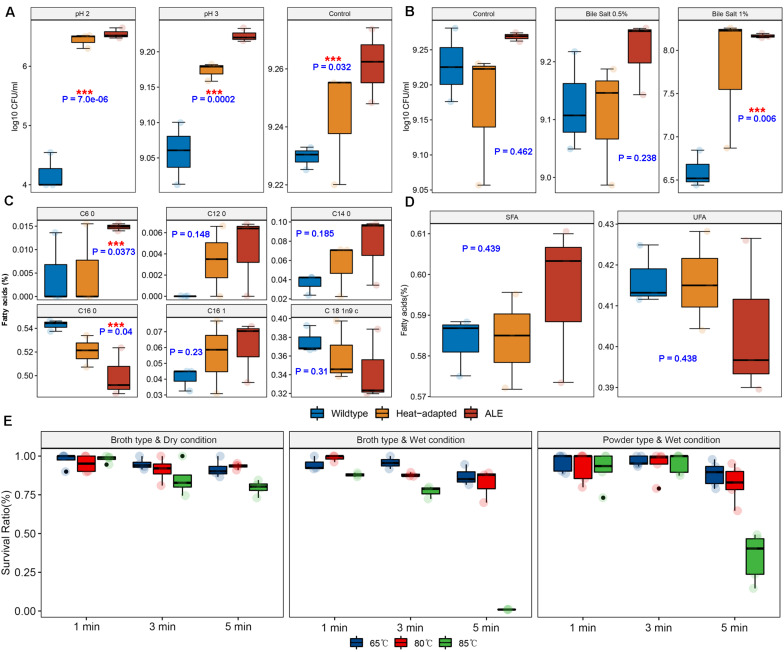
Investigation of survival rate under various conditions. *P*-values of the contrast test between WT vs. ALE group in one-way ANOVA model. **(A)** Change in survival rate under various acidity conditions. **(B)** Change in survival rate under various bile salt conditions. **(C,D)** Comparison of fatty acid compositions among BIOPOP-3 WT, heat-adapted, and ALE strains. **(E)** Tolerance limits for various conditions including packaging (broth/powder), humidity (wet/dry), temperature and exposure times important for feed manufacturing processes.

We hypothesized that BIOPOP-3 ALE could have improved resistances to other stresses such as acid and bile salts. In different acidic conditions, BIOPOP-3 ALE showed better survival rates than the WT and heat-adapted strains (Figure 5A). The BIOPOP-3 ALE also showed improved survival rate compared to WT, under different concentrations of bile salt (Figure 5B). Particularly, the survival rate was significantly greater (P: 0.006) than WT at the bile salt concentration of 1%. These results of two different stress conditions show that the evolved BIOPOP-3 strain acquired improved acid and bile stress resistance as cross protection. We further compared the fatty acid contents, assuming that the BIOPOP-3 ALE strain could change the content of saturated fatty acids for robust cell walls. Statistically significant differences were observed for strains only in caproic acid (C6:0) and hexadecanoic acid (C16:0) at 5% significance level (Figure 5C). Although it may not be statistically significant, it was observed that fatty acid contents were greater in ALE than WT for caproic acid (C6:0), lauric acid (C12:0), myristic acid (C14:0), and palmitoleic acid (C16:1). In contrast, higher contents of hexadecanoic acid (C16:0) and oleic acids (C18:1) were observed in WT. When we compared total fatty acid compositions of WT and ALE strains, the total saturated fatty acids (SFAs) concentration of BIOPOP-3 ALE strain was the highest (59.56 ± 1.12%) among all strains. Also, the total SFAs concentration of heat-adapted strain was 58.41 ± 0.69% which was higher than WT strain (58.34 ± 0.42%). In contrast, the total unsaturated fatty acids (UFAs) concentration of 41.66 ± 0.42%, 41.59 ± 0.69%, and 40.44 ± 1.12% were observed for WT, heat-adapted and ALE strains, respectively. Even when the fatty acid contents were divided into SFAs and UFAs, no statistical significance was observed, but linear patterns could be observed (Figure 5D). The relatively strong resistance to various stresses can be a benefit to probiotic strains used for the animal feed production process. We further experimented on additional conditions including packaging (Broth/Powder) and humidity (Dry/Wet). At 65 and 80°C, the survival rate of BIOPOP-3 ALE strain was maintained over 80% in most experimental conditions (Figure 5E). However, we found that the survival rate dramatically decreased under broth type and wet condition (0.98 ± 0.14%) and powder type and wet condition (34.91 ± 2.65%) at 85°C for 5 min. We further evaluated the long-term storage stability of animal feed mixed with the freeze-dried ALE strain and showed that it can be stably stored for 6 months (Supplementary Figure 2).

### Genetic Feature of BIOPOP-3 ALE Strain

**FIGURE 6:**
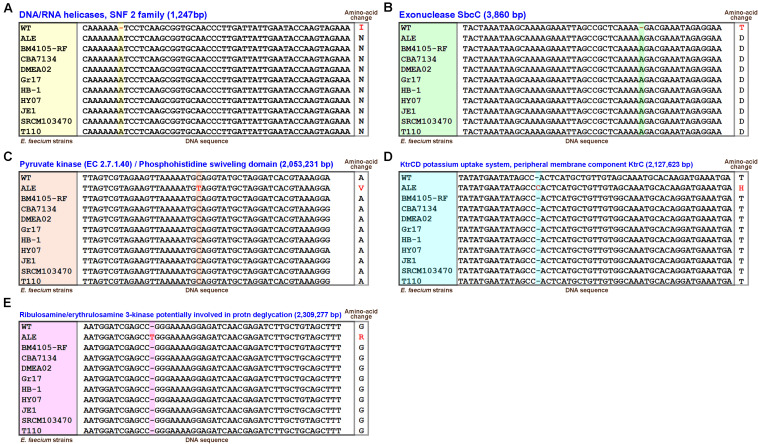
The 5 variants called by comparing BIOPOP-3 WT and ALE strains. **(A)** DNA/RNA helicase, **(B)** exonuclease SbcC, **(C)** pyruvate kinase/Phosphohistidine swiveling domain, **(D)** KtrCD potassium uptake system, peripheral membrane component KtrC, and **(E)** ribulosamine/erythrulosamine 3-kinase. The genotypes of nine *E. faecium* strains that were evolutionarily close to BIOPOP-3 were also presented.

We sequenced and assembled whole genome sequence of BIOPOP-3 ALE and compared it to the genome of the BIOPOP-3 WT to identify the genetic markers responsible for the heat resistance. A total of five mutations, consisting of 1 single nucleotide variation (SNV), and 4 single-nucleotide indels were identified (Figure 6). These genetic markers were technically verified for their genotypes using re-sequencing method. We also compared the genotypes of nine *E. faecium* strains similar to the BIOPOP-3 WT (Figures 2A–C) to examine these five genetic markers. A SNV variant in the region encoding for the pyruvate kinase was found, substituting nucleotide from C (Cytosine) to T (Thymine), causing change in amino acid from A (Alanine) to V (Valine) in the BIOPOP-3 ALE (Figure 6C). The other four genetic variations were found to be indels causing frameshift mutations. The genetic variants in the regions encoding for DNA/RNA helicase protein and exonuclease SbcC gene were specifically found in the BIOPOP-3 WT (Figures 6A,B). The other three genetic variants were specifically found in the BIOPOP-3 ALE (Figures 6C–E). These variants are the candidate markers for the superior stress resistance of the BIOPOP-3 ALE.

## Discussion

We first conducted a genome study to characterize the WT strain derived from fermented dairy product. We analyzed whether mobile elements such as prophage, CRISPR region and GI exist in the BIOPOP-3 genome. Genetic elements that have been shifted horizontally from other species can play important roles such as improved bacterial fitness, genomic stability, and strain-specific effects (Hacker and Carniel, 2001). However, it can adversely affect the host and become a pathogen (Roberts and Kreth, 2014). In this study, we identified 5 prophage regions, 9 GIs and 1 CRISPR region in the chromosome (Figures 1C,D). There were no virulence factors or resistance genes in GIs. The absence of virulence-related genes in the genome is one of the desirable properties expected from probiotic strains.

We characterized bacteriocins that are generally known to inhibit or kill other harmful microorganisms in the genome of BIOPOP-3 WT. This feature recently attracted attention as a natural antibiotic (Silva et al., 2018). We confirmed that the BIOPOP-3 strain possesses bacteriocin-related genes, including sactipeptide and enterolysin A (Figure 1D). Sactipeptide is a ribosomally synthesized peptide that contains intramolecular linkages between sulfur of cysteine residues and the α-carbon of another residue by post-translational modifications (Arnison et al., 2013). It is class I bacteriocin and shows some antimicrobial activity. Enterolysin A is class III bacteriocin first identified from an *Enterococcus* and inhibits growth of other bacteria by degrading of the cell wall (Nilsen et al., 2003). We identified enterolysin A in plasmid, but it was found with the identity of only 36.36%. Based on this result, it is difficult to conclude if this gene still function as a bacteriocin. Additionally, TIIIPKS was identified in BIOPOP-3 (Supplementary Table 3). TIIIPKS is a gene cluster can be detected in bacteria, as well as fungi and plants (Katsuyama and Ohnishi, 2012). In case of bacteria, it is known to be related to the defense system (Zeng et al., 2012) and for its activities such as antibiotics, anticancer, immuno-repressive and cholesterol lowering effects (Shimizu et al., 2017). Discovery of bacteriocin and TIIIPKS in BIOPOP-3 strain could mean that this strain could have antibacterial activity with pharmacological advantages which is beyond the scope of the current study.

In the comparative genomic analysis with 112 *E. faecium* strains, we found two clusters; one comprised of nine strains with high similarity to BIOPOP-3 and the other cluster of remaining 102 strains (Figure 2A). Most strains of this *E. faecium*, including BIOPOP-3, were isolated from fermented foods, except for the two strains, CBA7134 (NCBI accession number: PRJNA428410) and HB-1 (NCBI accession number: PRJNA546487) isolated from feces (Figure 2B). We speculated that these strains have slightly different genetic backgrounds as they adapt to different habitats (Zhong et al., 2019). In the COG functional analysis, we found significant difference between the two clusters in three COG categories, category X (Mobilome), category C (Energy production and conversion), and category U (Intracellular trafficking, secretion, and vesicular transport) while no significant differences were found in most functional categories (Figure 2C). A previous study has shown that stress can be induced by proteins which function as stabilizing the energy and metabolism supply of cells. Also, the study suggested these proteins are associated with stress resistance (Macario et al., 2006). The proteins which belong to significantly different COG categories may be one underlying cause of high stress resistance observed in BIOPOP-3 strain. We further investigated the pan-genome features to understand bacterial evolution, adaptation, and population structure for 10 *E. faecium* strains including BIOPOP-3 (Mira et al., 2010). The pan-genome contained a family of 3,915 genes, of which 1,959 genes were conserved across strains (Figures 2D,E). As the number of comparative strains increased, the size of the pan genome gradually increased, while the size of the core genome stabilized similar to the previous study of the *E. faecium* (Mikalsen et al., 2015; Ghattargi et al., 2018). Based on this result, we found an open pan-genome structure in 10 adjacent *E. faecium* strains including BIOPOP-3.

Generally, environmental stressors cause various challenges to microorganisms. The stress resistance of the probiotics is an important factor to consider in the manufacture and storage of probiotic products (Gueimonde and Sánchez, 2012). Previous studies reported that robust heat-resistant strains have the ability to maintain its cell size at high temperature (Gibson, 1973; Kilstrup and Hammer, 2000) allowing to maintain vital functions. Other studies have also highlighted advantages of the heat-resistant strains such as faster lactate production, less by-product formation, reduced risk of phage infection and high growth rate at higher temperatures (Hofvendahl et al., 1999; Adamberg et al., 2003; Chen et al., 2015). Therefore, the heat-resistant strains are of great interest to many industries. In this study, a modified ALE method consisting of two steps was applied to develop the robust heat-resistant strain (Figure 3). The first step of ALE demonstrates the potential of the method in inducing heat resistance and from this we also found the thermal threshold of BIOPOP-3, 75°C (Figure 4A). We additionally carried out new ALE experiment as the second step, which induced improved heat resistance and increased thermal threshold. The superiority of the two-step method was verified through comparison between BIOPOP-3 ALE, the product of the proposed two-step ALE method, with WT and heat-adapted strain which is the product of the first step of ALE method (Figure 4). The heat resistance of BIOPOP-3 ALE was demonstrated through a number of experiments considering various conditions in this study. In the second step of the ALE method, we observed two major changes in the survival rate on day 7 and day 13 in the 25 days of ALE procedure (Figure 4B). It is possible that the attempts to further improve the heat resistance of the evolving strain failed for initial experiments but was successful on day 7 and day 13.

In previous studies, heat stress has been shown to induce cross-protection against oxidative stress (Farr and Kogoma, 1991; Dowds, 1994). Based on this finding, we hypothesized that the improved heat resistance of the BIOPOP-3 ALE could contribute to cross-protection. In order for probiotics to be applied in the industrial field, they must withstand multiple stress (Henriksson et al., 1999). The BIOPOP-3 ALE strain did not show statistically significant increase in multiple resistance over other strains presumably due to small sample size (Figures 5A,B); however, a pattern of increase in survival rates could be observed from WT to heat-adapted strain and from the adapted strain to the ALE. Similar results were found for the contents of fatty acids (Figures 5C,D). On average, the total SFAs content showed positive correlation with the heat resistance, while the total UFAs content showed negative correlation. Previous studies have shown that increasing the contents of SFA could result in decrease of membrane fluidity, thereby increasing heat resistance which is consistent with the finding in this study (Abee and Wouters, 1999; Álvarez-Ordóñez et al., 2008; Shin et al., 2018).

To fully understand the genetic background of the BIOPOP-3 ALE strain underlying the excellent multi-stress resistance, we compared its whole genome sequence with that of BIOPOP-3 WT strain and found the five genetic variants located in the genic regions (Figure 6). We identified a gene annotated with “DNA/RNA helicase” is related with a DEAD-box RNA helicase, SNF2 subfamilies (Figure 6A). DEAD-box proteins are conserved RNA helicases associated with RNA metabolism and cellular processes (Cordin et al., 2006; Owttrim, 2006). Recently studies showed that DEAD-box RNA helicases were related to multiple stress resistance including the resistance toward low temperature in *Listeria monocytogenes* (Azizoglu and Kathariou, 2010), and the resistance toward alkali and heat stress in *Bacillus cereus* (Pandiani et al., 2011). We also identified a genetic variant located in the exonuclease SbcC (Figure 6B). A previous study has shown that SbcC contributes to survival against bile stress in *Enterococcus faecalis* (Breton et al., 2002). These two genetic variants were specifically found in the BIOPOP-3 WT. In addition, we found three genetic variants in which genetic mutations occurred in the BIOPOP-3 ALE strain only. The first of the candidate variants was found in Pyk (Figure 6C). This genetic variant specifically causes an amino acid substitution in only BIOPOP-3 ALE strain. According to a previous study, overproduction of Pyk increased acid and bile resistance in *Lactococcus lactis* (Zhai et al., 2015). It was also confirmed that Pyk was induced in *Lactobacillus acidophilus* during low temperature adaptation (Wang et al., 2005). In addition, previous studies show that the use of pyruvate in stress conditions changes to induce into fatty acid biosynthesis instead of other pathways such as butanoate metabolism and lactic acid synthesis in *L. lactis* and *Lactobacillus bulgaricus* (Ramos et al., 2004; Fernandez et al., 2008). Thus, we predict that the modification of the Pyk gene might induce SFA synthesis by rerouting pyruvate metabolism to fatty acid biosynthesis. Another candidate variant of BIOPOP-3 ALE strain was found in the KtrCD gene related to potassium uptake system (Figure 6D). Previously, it has been reported that disruption of KtrCD in *Bacillus subtilis* decreases resistance to osmotic stress (Holtmann et al., 2003). It is also well known that resistance of osmotic stress is associated with heat resistance, as it prevents expansion that occurs at high temperatures, allowing cells to maintain membrane integrity and intracellular environment (Chen et al., 2015). Combining the results of these two previous studies reveals that the genetic variant located in the KtrCD could contribute heat resistance mechanism. The last candidate variant identified was in ribulosamine/erythrulosamine 3-kinases (Figure 6E). Unfortunately, to the best of our knowledge, there is no existing study where ribulosamine/erythrulosamine 3-kinase was associated with stress resistance.

These findings support that the identified genetic variants in this study may be candidate markers associated with the improved stress resistance of BIOPOP-3 ALE strain. However, this study has several limitations. Although the two-step ALE method produced better heat resistance in *E. faecium* strain than the conventional ALE method, it was not applied to other strains. We expect to apply this approach in multiple strains in future studies. In addition, five genetic marker candidates of heat resistance have been proposed, but no experimental validation has been carried out. We expect to perform further research on this to explore the underlying mechanisms via genetic engineering techniques. Finally, over 10^9^ strains cultured based on two-step ALE were pooled and sequenced with PacBio. Although genotypes of all subclones could not be identified, the five candidate makers found in this study could be more reliable because they are conserved in the entire population.

In summary, we isolated BIOPOP-3 WT from fermented dairy product and completed the full-length genome sequence. We compared it with the publicly available sequences and confirmed that BIOPOP-3 is an *E. faecium* strain. After that, we developed BIOPOP-3 ALE, which can withstand various process stresses than the wild-type version. We conducted experiments under various stress conditions, and in most cases, BIOPOP-3 ALE showed a significantly improved survival rate. We also completed the whole genome sequence of BIOPOP-3 ALE strain to identify genetic variants of ALE strain. As a result, a total of five candidate markers were found. In addition, by conducting additional experiments to determine the process limitations of BIOPOP-3 ALE strain, we demonstrated that the strain can be applied to feed production processing in the industrial field (Figure 5E). In addition, the two-step ALE method proposed by this study has been proven to increase the stability of strain and resistances to multiple stresses simultaneously in *E. faecium* strain. Based on these advantages, we expect the two-step ALE method and the BIOPOP-3 ALE strain to be actively applied in the industrial field.

## Data Availability Statement

The datasets presented in this study can be found in online repositories. The names of the repository/repositories and accession number(s) can be found in the article/ Supplementary Material.

## Author Contributions

HK and BM conceived the project and designed the ALE experiments. BM performed the total experiments, analyzed the data, and wrote the manuscript. MS is a collaborator in the data analysis and supervised in writing the manuscript. DY helped genome data analysis and in writing the manuscript. YL helped in writing the manuscript. All authors contributed to the article and approved the submitted version.

## Conflict of Interest

HK was employed by company with C&K Genomics Inc. The remaining authors declare that the research was conducted in the absence of any commercial or financial relationships that could be construed as a potential conflict of interest.

## Footnotes

1 http://www.ncbi.nlm.nih.gov/genome/genomes/871
