# Supplementary material for: Complete Genomic Analysis of Enterococcus faecium Heat-Resistant Strain Developed by Two-Step Adaptation Laboratory Evolution Method
Source: Front Bioeng Biotechnol. 2020 Jul 23;8:828. doi: 10.3389/fbioe.2020.00828 (PMC7391244; doi:10.3389/fbioe.2020.00828)
Supplement: Supplementary file 1 [file Data_Sheet_1.DOCX]

**[Supplementary Materials]**

**Complete genomic analysis of *Enterococcus faecium* heat-resistant strain developed by Two-step Adaptation Laboratory Evolution method**

**Supplementary Figures**

[Supplementary Figure 1. Predicted genomic regions related secondary metabolism. 2](#_Toc36809267)

[Supplementary Figure 2. Long-term storage stability. 3](#_Toc36809268)

**Supplementary Tables**

[Supplementary Table 1. Identified prophage regions of BIOPOP-3 wild type genome 4](#_Toc36809318)

[Supplementary Table 2. Identified CRISPR regions of BIOPOP-3 wild type genome 5](#_Toc36809319)

[Supplementary Table 3. Predicted regions related secondary metabolism 6](#_Toc36809320)

[Supplementary Table 4. Predicted antibiotic resistance gene from CARD database. 7](#_Toc36809321)

[Supplementary Table 5. Comparison of COG functional terms between outgroup and ingroup *E. faecium strains* similar to BIOPOP-3. 8](#_Toc36809322)

**Supplementary Figures**


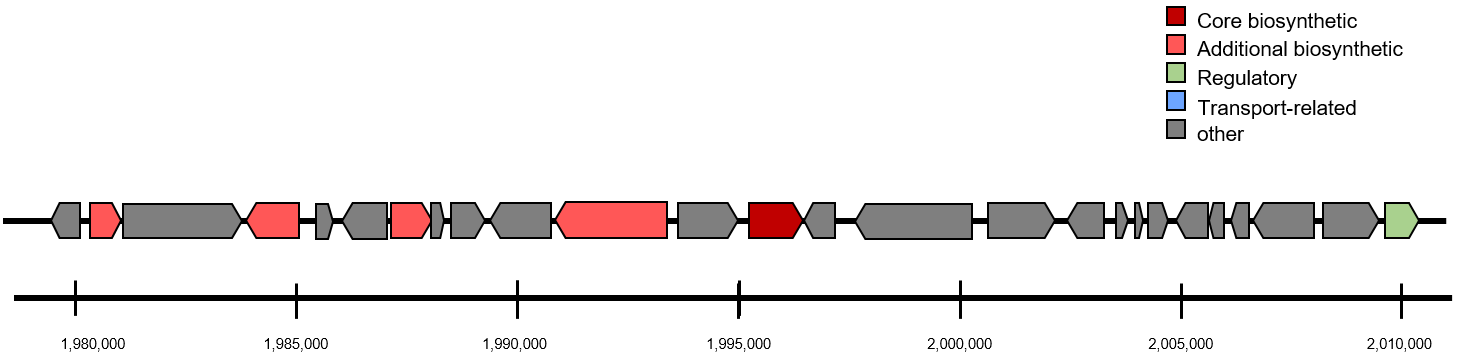


Supplementary Figure 1. Predicted genomic regions related secondary metabolism.

**Log CFU/ml**

**Time** (month)


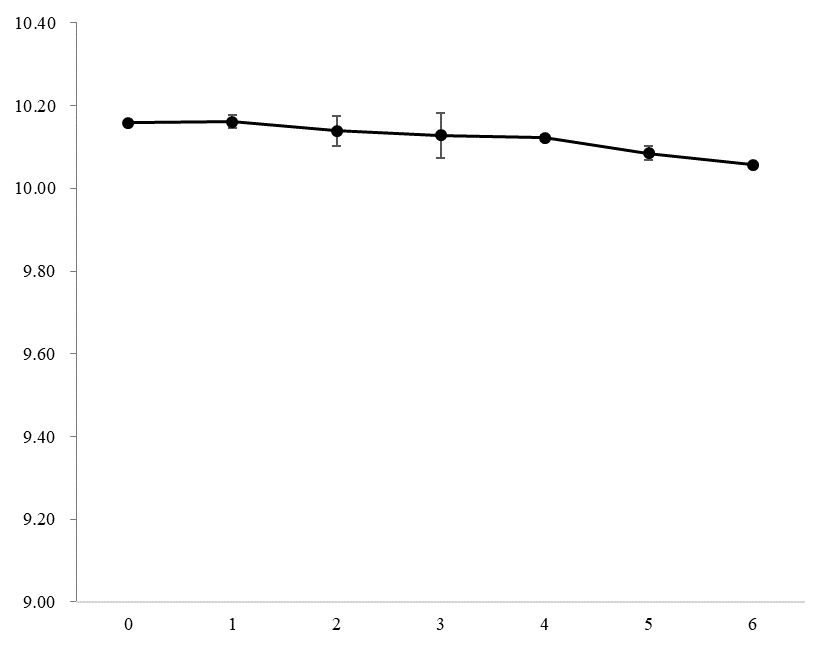


Supplementary Figure 2. Long-term storage stability.

**Supplementary Tables**

Supplementary Table 1. Identified prophage regions of BIOPOP-3 wild type genome

| **Type** | **Region length (Kb)** | **Region Position** | **Completeness** | **Score** | **GC contents (%)** | **Phage hit proteins** |
| --- | --- | --- | --- | --- | --- | --- |
| Chromosome | 8.8 | 1017222-1026058 | Incomplete | 30 | 33.8 | 8 |
|  | 11.6 | 1305178-1316874 | Incomplete | 60 | 30.9 | 16 |
|  | 50.5 | 2468514-2519059 | Complete | 140 | 37.5 | 61 |
|  | 23 | 2528000-2551002 | Complete | 140 | 39.8 | 27 |
|  | 23.1 | 2552725-2575878 | Incomplete | 30 | 37.0 | 33 |
| Plasmid | 39.1 | 3622-42759 | Complete | 140 | 34.1 | 24 |
|  | 7.7 | 31445-39182 | Incomplete | 60 | 36.2 | 9 |

Supplementary Table 2. Identified CRISPR regions of BIOPOP-3 wild type genome

| **Element** | **Genome** | **Start** | **End** | **Spacer /Gene** | **Repeat consensus /**  **cas genes** | **DR* length** | **Conservation DR*** |
| --- | --- | --- | --- | --- | --- | --- | --- |
| CRISPR | Chr. | 1956873 | 1956966 | 1 | TGAGGAAGAAGGTGTTGTTTCTGCTGCA | 28 | 96.43 % |
| CRISPR | Plasmid | 102360 | 102488 | 2 | GGTATGATTTTCATACCCTCTTGA | 24 | 88.52 % |

(*) DR**:** represents interrupted direct repeats

Supplementary Table 3. Predicted regions related secondary metabolism

| **Location** | **length** | **Legend** | **Function** | **E-value** |
| --- | --- | --- | --- | --- |
| 1980444 - 1981163 | 720 | Additional biosynthetic | alpha/beta hydrolase fold | 1.70E-21 |
| 1983969 - 1985156 | 1188 | Additional biosynthetic | GTP-binding protein LepA | 2.80E-106 |
| 1987247 - 1988098 | 852 | Additional biosynthetic | aldo/keto reductase family oxidoreductase | 1.20E-78 |
| 1990959 - 1993400 | 2442 | Additional biosynthetic | hydroxymethylglutaryl-CoA reductase | 8.00E-177 |
| 1995268 - 1996422 | 1155 | Core biosynthetic | T3PKS, Type III Polyketide Synthase | 4.5e-169 |
| 2009521 - 2010240 | 720 | Regulatory | GntR family transcriptional regulator | 1.30E-73 |

Supplementary Table 4. Predicted antibiotic resistance gene from CARD database.

| **RGI**  **Criteria** | **ARO Term** | **Start** | **SNP** | **Detection Criteria** | **AMR Gene Family** | **Drug Class** | **Resistance Mechanism** | **% Identity of Matching Region** |
| --- | --- | --- | --- | --- | --- | --- | --- | --- |
| Strict | AAC(6')-Ii * | 2390770-2391318 | - | protein homolog model | AAC(6') | aminoglycoside antibiotic | antibiotic inactivation | 98.9 |
| Strict | eatAv ^ | 969604 - 971106 | T -> I (450) | protein variant model | ABC-F ATP-binding cassette ribosomal protection protein | macrolide antibiotic, lincosamide antibiotic, streptogramin antibiotic, tetracycline antibiotic, oxazolidinone antibiotic, phenicol antibiotic, pleuromutilin antibiotic | antibiotic target protection | 96.2 |

*AAC(6')-Ii : neomycin, dibekacin, amikacin, sisomicin, netilmicin, kanamycin A, tobramycin, isepamicin, arbekacin, gentamicin B, plazomicin

^eatAv : pleuromutilin

Supplementary Table 5. Comparison of COG functional terms between outgroup and ingroup *E. faecium strains* similar to BIOPOP-3.

| COG | Function | P-value | FDR adjusted P |
| --- | --- | --- | --- |
| G | Carbohydrate transport and metabolism | 0.480978 | 0.58786228 |
| J | Translation, ribosomal structure and biogenesis | 0.636768 | 0.667090487 |
| K | Transcription | 0.378284 | 0.489543497 |
| S | Function unknown | 0.002257 | 0.027395641 |
| R | General function prediction only | 0.268258 | 0.368855075 |
| E | Amino acid transport and metabolism | 0.110576 | 0.224948308 |
| M | Cell wall/membrane/envelope biogenesis | 0.014902 | 0.065567947 |
| L | Replication, recombination and repair | 0.124777 | 0.224948308 |
| P | Inorganic ion transport and metabolism | 0.018403 | 0.067478299 |
| T | Signal transduction mechanisms | 0.113165 | 0.224948308 |
| F | Nucleotide transport and metabolism | 0.02575 | 0.076368266 |
| I | Lipid transport and metabolism | 0.143149 | 0.224948308 |
| O | Posttranslational modification, protein turnover, chaperones | 0.02777 | 0.076368266 |
| V | Defense mechanisms | 0.262659 | 0.368855075 |
| C | Energy production and conversion | 0.006512 | 0.035815969 |
| H | Coenzyme transport and metabolism | 0.13628 | 0.224948308 |
| X | Mobilome: prophages, transposons | 0.002491 | 0.027395641 |
| D | Cell cycle control, cell division, chromosome partitioning | 0.063706 | 0.155725315 |
| U | Intracellular trafficking, secretion, and vesicular transport | 0.006091 | 0.035815969 |
| Q | Secondary metabolites biosynthesis | 0.604086 | 0.664494271 |
| N | Cell motility | 0.842653 | 0.842652767 |
| W | Extracellular structures | 0.56703 | 0.656560783 |
